# Role of non-canonical post-translational modifications in gastrointestinal tumors

**DOI:** 10.1186/s12935-023-03062-x

**Published:** 2023-09-30

**Authors:** Yihong Liu, Jingwei Liu, Na Peng, Shuangshuang Hai, Shen Zhang, Haibo Zhao, Weixin Liu

**Affiliations:** 1 Department of Gastroenterology, the First Affiliated Hospital of China Medical University, 155# North Nanjing Street, Heping District, Shenyang City, 110001 Liaoning Province China; 2 Department of Anus and Intestine Surgery, the First Affiliated Hospital of China Medical University, Shenyang, Liaoning Province China; 3 Department of Gastroenterology, the Fourth Affiliated Hospital of China Medical University, Shenyang, Liaoning Province China

**Keywords:** Gastrointestinal tumors, PTM, Carcinogenesis

## Abstract

Post-translational modifications (PTMs) of proteins contribute to the occurrence and development of tumors. Previous studies have suggested that canonical PTMs such as ubiquitination, glycosylation, and phosphorylation are closely implicated in different aspects of gastrointestinal tumors. Recently, emerging evidence showed that non-canonical PTMs play an essential role in the carcinogenesis, metastasis and treatment of gastrointestinal tumors. Therefore, we summarized recent advances in sumoylation, neddylation, isoprenylation, succinylation and other non-canonical PTMs in gastrointestinal tumors, which comprehensively describe the mechanisms and functions of non-classical PTMs in gastrointestinal tumors. It is anticipated that targeting specific PTMs could benefit the treatment as well as improve the prognosis of gastrointestinal tumors.

## Introduction

Protein post-translational modifications (PTMs) are biochemical mechanisms by which amino acid residues in proteins are covalently modified. Until now, over 400 kinds of PTMs of proteins have been identified, of which canonical PTMs include acetylation, ubiquitination, phosphorylation, methylation and so on. Through adding small chemical groups or small proteins, inactive precursor proteins change into functional mature proteins of diverse properties. Emerging evidence suggested that PTMs of proteins participate in tumorigenesis, progression, prognosis and treatment of cancers [1–3]. In recent years, multiple non-canonical protein PTMs were identified including SUMOylation, isoprenylation and palmitoylation, which have also been found to be involved in various aspects of tumors.

Gastrointestinal tumors continue to be one of the most common tumors worldwide and pose a significant global public health burden, with their high rates of incidence and mortality. A variety of factors such as genetics, diet, infection, and epigenetic changes give rise to the carcinogenesis and development of gastrointestinal tumors [4, 5]. Recent investigations indicate significant involvement of non-canonical protein PTMs in gastrointestinal tumors. For example, SUMO modification of annexin A4 (ANXA4), a calcium-binding protein located in the gastrointestinal tract, increases its expression and promotes epithelial-mesenchymal transition (EMT) of tumor cells [6]. In human colorectal tissues, the K420 site lysine crotonylation of alpha-enolase (ENO1) enhances its enzyme activity, thus promoting tumorigenesis and aggressiveness [7]. NEDD8-Activating Enzyme inhibitor MLN4924 has been reported to suppress the growth and migration of gastric cancer cells by blocking the neddylation of the E3 ubiquitin ligase CRL [8]. In addition, CPT1A promotes lysine residue k222 succinylation of lactate dehydrogenase A (LDHA), which is associated with tumor cell proliferation and poor prognosis of patients [9].

In this review, we summarized recent advances in the studies investigating the role of non-canonical PTMs including SUMOylation, isoprenylation, neddylation, citrullination, succinylation and other modifications in the occurrence, development, treatment and prognosis of gastrointestinal tumors. It is anticipated that understanding and targeting these novel protein PTMs could benefit therapeutic strategies for gastrointestinal tumors.

## SUMOylation

**Fig. 1:**
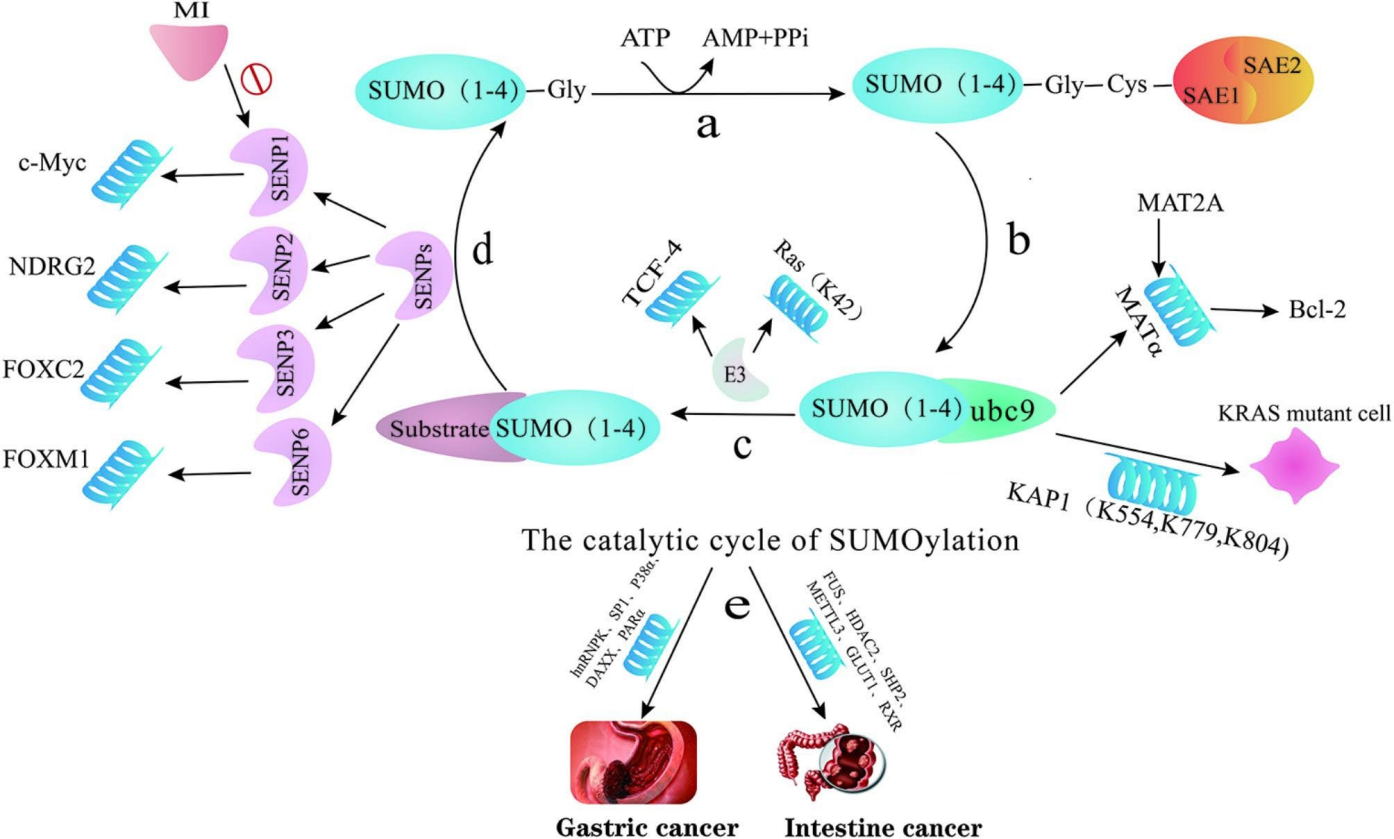
Process of Sumoylation cycle. **a**. activation: ATP-dependent enzyme E1 enzyme (SAE1/SAE2) acts to link the Gly end of SUMO at the Cys end of E1 enzyme. **b**. conjugation: activated SUMO is transferred to E2 enzyme (ubc9), which is linked to the Cys end of ubc9. **c**. ligation: E3 acts to recognize the Lys end of the substrate and facilitate the SUMOylation. **d**. de-SUMOylation: de-SUMOylation occurs by the action of SENPs, and SUMO is removed from the target protein. **e**. Human prone to gastrointestinal tumors by promoting/inhibiting SUMO modification

SUMOylation is a dynamic and reversible post-translational modification mediated by E3 ligases, dimeric SUMO E1 SAE1/UBA2 and a single E2 ubc9, often with the involvement of protein inhibitor of activated STAT (PIAS) family members, and Ran binding protein 2 (RanBP2) and SUMO protease SENPs (Sentrin-specific proteases). Small ubiquitin-like modifier (SUMO) is a highly conserved 11 KDa protein, of which four isoforms of SUMO are known, including SUMO1, SUMO2/3 (SUMO2 and SUMO3 have 95% sequence homology) and SUMO4. SUMOylation is involved in multiple biological processes such as genomic stability, cell differentiation, DNA damage response, cancer cell proliferation and invasion [10, 11] (Fig. 1).

### SENP-mediated SUMOylation

SENPs are SUMO-specific proteases, originally known as Sentrin-specific proteases, that remove SUMO from target proteins. There are six members of the mammalian SENP family, which can be bifurcated into three subfamilies, SENP1 and SENP2 as one family, SENP3 and SENP5 as one family, and the remaining SENP6 and SENP7 as one family [12, 13].

It was found that SENP1 was overexpressed in colon cancer tissues, and silencing SENP1 expression could inhibit the growth of colon cancer cells by arresting the cells in G1 phase [14]. Momordin Ic(MI), an emerging SENP1 inhibitor, can act as an anti-cancer drug by inhibiting the de-SUMOylation of c-Myc and reducing the level of c-Myc protein, which lead to cell cycle arrest and apoptosis of tumor cells [15]. In addition, SENP2 can mediate the de-SUMOylation and stabilization of N-myc downstream regulatory gene 2 (NDRG2) in gastric cancer, thereby exerting its anti-tumor effect [16].

The transcription factor forkhead box proteins play an essential role in regulating the cell cycle and promoting the development of gastric cancer. SNEP3 activates the transcriptional activity of forkhead box protein C2 (FOXC2) and increases the expression of N-cadherin through the deSUMOylation of FOXC2, which activates the transformation of epithelial mesenchyme as well as enhances the invasion and metastasis of gastric cancer cells [17]. The transcription factor forkhead box protein M1 (FOXM1) has been suggested to promote tumor formation by regulating cell cycle. SENP6 activates and stabilizes FOXM1 by catalyzing the de-SUMOylation of FOXM1, which leads to successful mitosis and promotes the growth of gastric cancer cells [18].

### Non-coding RNAs-regulated SUMOylation

LncRNAs refer to non-coding RNA with a length of more than 200 nucleotides [19]. CircRNAs are non-coding RNA with ring stricture, which stably express and regulate multiple biological processes [20]. Both lncRNAs and circRNAs have been found to regulate SUMOylation in gastrointestinal tumors.

In gastric cancer, LncRNA SDCBP2-AS1 blocks the SUMOylation of hnRNPK (heterogeneous nuclear ribonucleoprotein in K) via binding to the KH domain of hnRNPK, which destroys the stability of β-catenin and promotes its degradation, thus inhibiting the growth and migration of gastric cancer cells [21]. SUMOylation inhibition of SP1 increases its expression level, which promotes the expression of LncRNA SNHG17 and alters the miR-23b-3p/Notch2 pathway, thus promoting the occurrence and development of gastric cancer [22]. Besides, SUMO1 pseudogene 3 (SUMO1P3), a member of the SUMO pseudogene family that expresses lncRNA, is up-regulated in gastric cancer, which correlated with tumor size, differentiation, metastasis and invasion [23].

GAL (GLUT1 associated LncRNAs) is the lncRNA associated with colorectal liver metastasis (CRLM). In CRLM tissues, the expression of GAL is up-regulated to promote SUMOylation of GLUT1 protein, thus promoting metastasis of colorectal cancer by preventing the degradation and enhancing the stability of GLUT1 [24]. RMST, a proliferation and apoptosis-related lncRNA, binds with FUS located in the nucleus and enhances SUMO1 modification at the K333 site of FUS, thereby enhancing the stability of the complex, which promote mitosis and migration of colon cancer cells [25]. In addition, circ-GALNT16 can inhibit SENP2-mediated hnRNPK de-SUMOylation by binding to the KH3 domain of hnRNPK and inhibit the progression of colorectal cancer [26].

### Drug resistance-related SUMOylation

At present, the major treatment of gastrointestinal tumors is surgical treatment supplemented by chemoradiotherapy. For patients in advanced stages, radiotherapy and chemotherapy remain the main treatments. However, many drugs are prone to drug resistance in clinical practice [27]. Recent studies demonstrated that SUMOylation contributes to drug resistance in gastrointestinal tumors.

The expression of methyltransferase-like 3 (METTL3) is increased through SUMO1 SUMOylation modification in colorectal cancer, and increased METTL3 promotes the expression of ABCC1 protein, thus leading to drug resistance of colorectal cancer cells to chemotherapy [28]. The SUMOylation of IGF1R allows nuclear translocation of the transcriptional cofactor IGF1R, which is associated with drug resistance in colorectal cancer [29]. In addition, SUMOyaltion of deacetylase 2 (HDAC2) can protect colon cancer cells from chemotherapeutic-induced genotoxic stress by activating kinase RSK1 and enhancing the regulatory transcription of nuclear factor-KB (NF-KB), so high levels of HDAC2 correlated with chemotherapeutic-drug resistance in colon cancer [30].

Topoisomerase I (Popo-I) inhibitor irinotecan (CPT-11) and cisplatin (DDP) are commonly used in the treatment of gastrointestinal tumors [31]. In drug-resistant cells of colorectal cancer, the aberrantly expressed SENP1 could increase the SUMOyaltion of HIF-α, which leads to the conversion of intracellular signal transduction pathway from Wnt/β-catenin to EGFR/IKKα/β/NF-kB, thus reducing the sensitivity of cancer cells to irinotecan [32]. Furthermore, in cisplatin-resistant gastric cancer tissues, it was found that low-SUMOylation of SP1 increased the expression of SP1 in gastric cancer tissues, and the increased SP1 promoted drug resistance of gastric cancer cells through the SNHG17/miR-23b-3p/Notch2 axis [22].

### Oxidative stress and adaptation to hypoxia

Various intracellular and extracellular stimuli are involved in the pathophysiological processes of different diseases, of which oxidative signal is closely correlated with the occurrence and development of various tumors [33].

An anoxic microenvironment is a challenge that tumors must adapt to and overcome in order to survive [34], and in the anoxic environment, gastric cancer cells adapt to anoxic conditions by upregulating lysine-specific demethylase 5B (KDM5B). By enhancing the SUMOylation of KDM5B mediated by SUMO3 and responsible for SUMO E3 ligase PIAS4, hypoxia promotes the stability of KDM5B, which is conducive to the response of tumor cells to anoxic environment [35]. In addition, ROS promoted SUMO1-mediated p38α SUMOylation at the K152 site by stabilizing E3 SUMO protein transferase PIASxα, which ensures the stability and nuclear translocation of p38α as well as promotes the metastasis of gastric cancer cells [36]. SENP3 has been reported as a specific protease sensitive to redox reactions in gastrointestinal tumors [37]. SENP3 enhances the transcriptional activity of FOXC2 by participating in de-SUMOylation of FOXC1 under oxidative stress, thereby promoting EMT [17]. In colorectal cancer, the SUMOylation of PIAS itself can also regulate the function and stability of the protein. PIAS promotes HIF-1’s SUMOylation-mediated hypoxia adaptation by interacting with the ODD domain of HIF-1 [35, 38].

### ubc9

Ubiquitin-coupled enzyme 9 (ubc9), an essential E2-coupled enzyme of SUMOylation, has been found to prolong tumor cell survival through the function of B-cell lymphoma 2 (Bcl-2) in diverse cancers [39].

Methionine adenosine transferase 2 A (MAT2A) encodes MATα2, which is highly expressed in colon cancer and regulated by the SUMOylation of SUMO1, SUMO2 and SUMO3 [40, 41]. MATα2 could interact with ubc9 to increase Bcl-2 protein [42]. It was found that the deficiency of ubc9 in mice promoted cell growth and the formation of polyps. In colon cancer, increased ubc9 suppressed effective SUMOylation and promoted tumorigenesis [43].

RAS, a signal transduction molecule downstream of growth factor receptors, regulates the biological function of cells through multiple pathways and is often accompanied by KRAS gene mutation in colorectal cancer [44]. The SUMO ligase ubc9 is necessary for KRAS, and the activity of transcriptional repressor KAP1 is enhanced in KRAS mutated cells after SUMOylation at K554, K779, and K804 [45].

### Cell cycle and cell proliferation

The occurrence and development of gastrointestinal tumors are complex processes with various aspects involved, one of which is the excessive proliferation of cells [46]. SUMOylation can regulate tumor cell cycle and thus regulate tumor cell proliferation.

When carcinogenic stress signals in the body respond, the scaffold protein IQCAP1 regulates alternative splicing(AS) related to the cell cycle of gastric cancer cells by influencing the SUMOylation of the AS regulatory protein hnRNPM, thus promoting the proliferation of tumor cells [47]. Moreover, in colorectal cancer, IQGAP1 promotes the proliferation and migration of tumor cells by promoting the phosphorylation of ERK, MEK, and AKT through SUMO1-mediated SUMOylation at the lysine K1445 [48].

V-maf musculoaponeurotic fibrosarcoma oncogene family protein B(MAFB), a member of the Maf transcription factor family [49], plays a critical role in colorectal cancer. Mediated by SUMO1, MAFB is SUMOylated at the lysine 32 site, which affects its binding to cell cycle factors such as CDK6 and regulates G1/S phase transition [50]. Thus, MAFB plays a crucial role in promoting tumor cell proliferation and inhibition of MAFB leads to cell cycle arrest. Momordin Ic (MI) demonstrated therapeutic potential by inhibiting the SENP1/c-MYC signaling pathway allowing colorectal cell cycle arrest in the G0/1 phase [15].

Inhibition of SUMO1-activating enzyme subunit 1 (SAE1) can block colon cancer cells in the G0/G1 phase [51]. At the same time, ubiquitin-like modifier activating enzyme 2 (UBA2), namely SUMO activating enzyme subunit 2 (SAE2), is a crucial component of SUMOylation E1 enzyme [52]. Although the specific mechanism of UBA2 and SUMOylation remains unclear, inhibition of de-SUMOylation activation of UBA2 can reduce the levels of cyclingB1, Bcl-2, DMD2, p-AKT and affect p53/DMD2/p21 signaling pathway, thus influencing the transitions of the colorectal cancer cell cycle from G1/S and G2/M [53]. In colorectal cancer, high UBA2 expression is positively correlated with EZH2 expression and unfavorable prognosis of patients. E2F1 can be modified by SUMO1 at Lys-226, and SUMOized E2F1 promotes the expression of EZH2 by enhancing its binding to the EZH2 promoter [54].

### CSC

Cancer stem cells (CSCs), also known as tumor-initiating cells, are one of the main causes of colorectal cancer recurrence and metastasis as well as poor therapeutic effect during radiotherapy and chemotherapy [55].

Many transcription factors, including the AP-2 transcription factor, regulate the EMT process and are regulated by SUMOylation [56]. The SUMOylation of TFAP2A (transcription factor activating protein 2α) occurs at lysine 10 [57], and the binding of TFAP2A to SUMO is inhibited by E1 and E3 SUMO inhibitors. The CSCs of colorectal cancer are CD44^+/hi^ALDH^+/hi^ cell population which are mediated by SUMO-unconjugated TFAP2A. SUMO inhibitors inhibit colorectal cancer growth, invasion, and metastasis by inhibiting the CSCs population through the inhibition of CD44 and MMP14 [58].

ALDH, which is highly expressed in colorectal cancer CSCs, is not only a marker but also important for biological functions such as maintenance and self-renewal of CSCs [59]. TRIM21 acts as the ubiquitin E3 ligase of Oct-1, and the SUMOylation enhances the ubiquitination and degradation of Oct-1 (ALDH transcription factor) and reduces its stability by regulating the expression of its transcription factor interferon regulatory factor 1 (IRF1) [60].

### TCF-4 and NPC

T cell factor 4 (TCF-4) can bind to nuclear pore complex (NPC) protein, which is associated with the Wnt/β-catenin signaling pathway involved in tumorigenesis of colorectal cancer. The SUMOylation of TCF-4 promotes its interaction with β-catenin, while RanBP2, an NPC protein that acts as SUMO E3 ligase, can regulate the SUMOylation of TCF-4 by using RanGAP1 as the substrate and mediated by SUMO1. In addition, the overexpression of NPC protein can promote TCF-4 transcriptional activity and the Wnt/β-catenin signaling pathway [61]. It has also been found that SUMOylation mediated by UHRF2 regulates the stability of TCF-4 protein, thereby maintaining the stability of the Wnt/β-catenin signaling pathway, which in turn regulates UHRF2 expression in CRC [62].

The accumulation of death domain-associated protein (DAXX) in the nucleus of tumors is associated with the poor prognosis of gastric cancer. As a substrate of SUMO2/3, DAXX modulates its level by binding to SUMO2/3, and it regulates the cytoplasmic distribution of DAXX and increases the stability of the protein by affecting the SUMOylation, which is catalyzed by RanBP2/RanGAP1 [63]. Ras protein, another common NPC protein, is subject to SUMO3-mediated SUMOylation at lysine K42 and influences the occurrence and migration of colorectal cancer via the Raf/MEK/ERK signaling axis [64].

### Others

A number of other researches have been conducted on the relationship between SUMOylation and gastrointestinal tumors. P53 is the modified substrate of SUMO1, and SUMO1-mediated SUMOylation leads to increased levels of the p53 protein in colon cancer patients, which is associated with poor prognosis of patients [65]. In colorectal cancer, the zinc-finger-containing transcription factor KLF5 promotes nuclear localization and regulates cell proliferation through SUMOylation of glutamic acid residues at positions 153 and 204, and stabilizing KLF5 in the nucleus by inhibiting its leucine-rich nuclear exit signaling (NSE) activity [66]. CBX4 rs77447679 gene polymorphism was positively correlated with the high risk of gastric cancer, while the CC genotype had a low risk of gastric cancer [67]. In CRC, anti-PD-L1 antibody combined with irradiation (IR) + ATR inhibitor (ATRi) can enhance the efficacy of the therapy, which is based on the mechanism of activating STRING signaling by promoting SUMOylation of SHP1 at the lysine 127 locus, thus inducing the expression of IFN-I-related genes and promoting immunotherapy [68].

In patients with gastric cancer, the high expression of TRIM28 is closely related to the low survival rate of patients, which may be related to the direct binding and stabilization of PD-L1 by inhibiting the ubiquitination of checkpoint protein PD-L1 in gastric cancer cells and promoting its SUMOylation [69].In gastric cancer cells, it was found that all-trans retinoic acid (ATRA) could enhance the SUMOylation of retinoic acid receptor α (RARα) and improve its stability, thus promoting the formation of RARα and PXRα heterodimers in the nucleus, which is favorable to its signal transduction [70]. NSUN1-7 is a nucleolar RNA methyltransferase highly expressed in gastric cancer and plays a vital role as a writer for RNA modification of 5-methylcytosine (m5C) [71]. SUMO2/3-mediated SUMOylation of NSUN1-7 regulates its protein stability and promotes its transportation to the nucleus, thus exerting a carcinogenic role dependent on m5C methyltransferase [72]. Ginkgo acid (GA), a natural component of ginkgo, nuts, and seed coat [73], inhibits the migration, proliferation, and EMT of gastric cancer cells by inhibiting SUMO1-mediated SUMOylation of IGF-1 and reducing the expression level of SNA12 and its binding to IGF-1 [74].

## Neddylation

Neddylation is a reversible post-translational modification of proteins catalyzed by NEDD8-activated enzyme E1 (NAE), Nedd8-coupled enzyme E2 (UBE2F, UBE2M) and substrate nedd8-E3 ligase, which plays a crucial role in various biological processes such as tumorigenesis, tumor microenvironment and apoptosis by binding the ubiquitin-like protein NEDD8 to lysine residues of substrate protein [75, 76].

### CRLs and CUL

E3 ubiquitin ligases, generally classified into scaffold type and thioester bond intermediate type according to the difference between RING domain 1 and HECT domain 2 [77], play an important role in the biological functions of gastrointestinal tumors by participating in neddylation modification.

Under the mediation of NEDD8 E3 ligase Mdm2, neddylation of Hu antigen R (HuR) at K283, K313 and K326 promotes the stability of HuR protein and stabilizes its localization in the nucleus, which promotes the proliferation of gastrointestinal cancer cells [78]. The most abundant member of E3 ubiquitin ligases is the Cullin-RING E3 ligases (CRLs), a kind of crucial catalytic enzyme activated by the substrate cullin(CUL) during the neddylation. The CUL family has a total of seven members including CUL1, CUL2, CUL3, CUL4A, CUL4B, CUL5, and CUL7 [79]. CUL plays an important linker role in CRLs, and the neddylation of CUL and abnormal activation of CRLs are closely associated with gastrointestinal tumors (Table 1).

**Table 1:** Information of cullin in gastrointestinal tumors

CUL	Neddylation status	Cancer type	Regulatory proteins(axis)	Effects	References
CUL1	Neddylation	Gastric cancer	P21, P27,CDT1	Inhibition of CUL1 suppress tumor cell proliferation and promote tumor cell apoptosis	[8, 76, 77]
CUL1	de-neddylation	SI-NETs	CSN5, DKK1, TAZ	De-neddylation of CUL1 affects tumor progression	[75]
CUL3	Neddylation	Gastric cancer	KEAP1-CUL3-Nrf2	Promoting CUL3 can inhibit cell growth and induce cell apoptosis	[78]
CUL4A	Neddylation	Gastric cancer	NF-KB	Inhibition of CUL4A can inhibit tumor migration	[79, 80]
CUL5	Neddylation	Gastric cancer	NOXA	Promoting CUL5 can inhibit cell growth and promote apoptosis	[81]
CUL5	Neddylation	Colorectal cancer	NOXA	Promoting CUL5 promotes tumor growth and leads to drug resistance	[74]

CSN5 may regulate the stability of DKK1 by the de-neddylation of Cullin-RING-ligase, while DKK1 influences the development of colorectal cancer by regulating the wnt/β-catenin pathway [80]. CUL1 regulates the biological function of gastric cancer cells through two pathways. On the one hand, inhibition of CUL1 can up-regulate P21 and P27, thus limiting the proliferation of gastric cancer cells [81]. On the other hand, it is interesting to note that inhibition of CUL1 could theoretically lead to the accumulation of tumor suppressor proteins such as CDT1 and promote DNA damage [8], but it has also been suggested that this could block the accumulation of cancer suppressor proteins [82]. Enhanced neddylation of CUL3 can inhibit the growth of gastric cancer cells by the KEAP1-CUL3-Nrf2 axis, and induce tumor cell apoptosis by producing more ROS [83]. CUL4A can induce increased expression of NF-kB [84], and neddylated CUL4A preferentially binds to DNA methyltransferase (DNMT3b), thus inhibiting the formation of chromatin and gastric cancer development [85].

NOXA, as a substrate for CUL5, underwent neddylation under the mediation of CUL5 and may induce apoptosis of gastric cancer cells [86]. It is noteworthy that, under ROS induction, the oligomeric peroxiredoxin PRDX1 could specifically enhance the interaction between CUL5 and NEDD8-coupled enzyme UBE2F, thereby enhancing the neddylation of NOXA and promoting protein degradation, which is beneficial for colorectal cancer growth and can partially explain the drug resistance of etoposide during the treatment of colorectal cancer as well [79].

### NAE modulators

NAE is a 110 kd heterodimer composed of amyloid beta precursor protein binding protein 1 (APPBP1) and UBA3 [87]. The first step catalyzed by NAE is to form AMP-NEDD8 complex by combing AMP and NEDD8, which makes AMP occupy the adenylated domain of NAE, and then carry out the following enzyme-linked cascade reaction [88]. Numerous studies have shown that a variety of tumors can be treated by inhibiting NAE, which makes NAE inhibitors a promising anticancer drug for gastrointestinal tumors.

#### NAE inhibitors: MLN4924; mitoxantrone; various derivatives

MLN4929, the first reported NAE inhibitor, has been approved for clinical trials in gastric cancer [89]. MLN4929 could specifically inhibit the formation of the SCF complex composed of SKP2, CUL1, and substrate recognition protein F-box protein by blocking the neddylation of CUL1. MLN4929 regulates the hydrolysis of proteins mediated by SCF and causes the accumulation of CRLs substrates, which can activate autophagy of gastric cancer cells as well as induce G2/M phase arrest and apoptosis. In addition, it can inhibit the distant metastasis of gastric cancer cells by up-regulating the epithelial biomarker E-cadherin and down-regulating the matrix metalloproteinase (MMP-9) through transcriptional activation [8, 90].

MLN4924 can induce the interaction between Mof4 family-associated protein 1 (MRFAP1) and cyclin-dependent kinase inhibitor 1B (P27) to enhance the stability of tumor suppressor and cell cycle regulator P27. Stable P27 plays a protective role by stabilizing mitochondrial membrane permeability. Therefore, the inhibition of MRFAP1 or P27 combined with MLN4924 can improve the therapeutic effect of MLN4924 on gastric cancer [90, 91]. High mutant phenotype tumors caused by DNA mismatch repair (dMMR), also known as microsatellite instability (MSI) tumors, accounting for a large proportion of gastrointestinal tumors [92]. It was found that MLN4924 could induce immunogenic cell death in the colorectal cancer cells by suppressing the clearance of MSI instability protein; in turn, the unstable protein could enhance the sensitivity of MLN4924 and thus produce an anti-cancer effect [93].

The NAE inhibitor mitoxantrone is also approved for clinical trials in advanced gastric cancer [88]. As an ATP-competitive NAE inhibitor, mitoxantrone has high selectivity and inhibits the binding of UBE2M (also known as UBC12) to NEDD8, meanwhile, it can promote the apoptosis of gastrointestinal tumor cells by inhibiting the degradation of neddylation substrate p53 [94, 95].

There were studies on inhibiting NAE activity and neddylation by synthesizing the 1,2, 4-triazine derivative V11 against gastric cancer cells. It was found that V11 inhibited neddylation by blocking the formation of UBC12-NEDD8, cullin3 and cullin1-NEDD8, thus inhibiting the growth of gastric cancer cells and inducing apoptosis [96]. The novel indole derivative V7 could also inhibit the coupling of UBC12 and NEDD8 by binding to NAE to inhibit the proliferation of gastric cancer cells and induce apoptosis by inhibiting neddylation and the MAPK pathway [97]. The cinnamyl piperidine derivative compound 4 g could selectively inhibit the neddylation of gastric cancer cells mediated by cullin1 and cullin3, and promote the apoptosis of gastric cancer cells induced by mitochondria and Nrf2-Keap1 signaling pathway, thus inhibiting the growth and metastasis of tumor cells [98].

#### NAE agonists

Currently, only a few studies have studied neddylation agonists for the treatment of gastrointestinal tumors, such as tertiary amide derivatives [99], while 1,2, 4-triazine-dithiocarbamate derivatives have been synthesized to be the agonists of neddylation by targeting NAE. Some new compounds K1-K13 are designed and synthesized, and it has been proved that the most effective compound for gastric cancer cells is K3, which can promote neddylation by targeting NAE1 to increase the degradation of YAP/TAZ proteins. K3 decreases the level of anti-apoptotic protein c-IAP1 and cleave PARP protein to induce apoptosis and inhibit the proliferation of tumor cells [100, 101].

### Others

It is demonstrated that DCUN1D1, as a part of the E3 complex of neddylation, is the target gene of non-coding RNA miR-520b. In colorectal cancer, miR-520b can inhibit the proliferation, metastasis and EMT of tumor cells by targeting DCUN1D1 [102]. By binding with NEEDD8, TRIM inhibits the activity of NF-kB and prevents the transfer of NF-kB from the cytoplasm to the nucleus; meanwhile, it can effectively prevent gastrointestinal carcinogenesis by promoting neddylation of IKKγ complex [103]. The ubiquitin ligase Smurf1 can affect the progression and the poor prognosis of colorectal cancer through the activation of canonical neddylation and its specific neddylation at the C426 site [104]. In addition, RRP9, as a substrate for Smurf1, promotes the processing of Pre-rRNA and the occurrence of colorectal cancer by interacting with Smurf1 to mediate neddylation at K221 [105].

## Isoprenylation

**Fig. 2:**
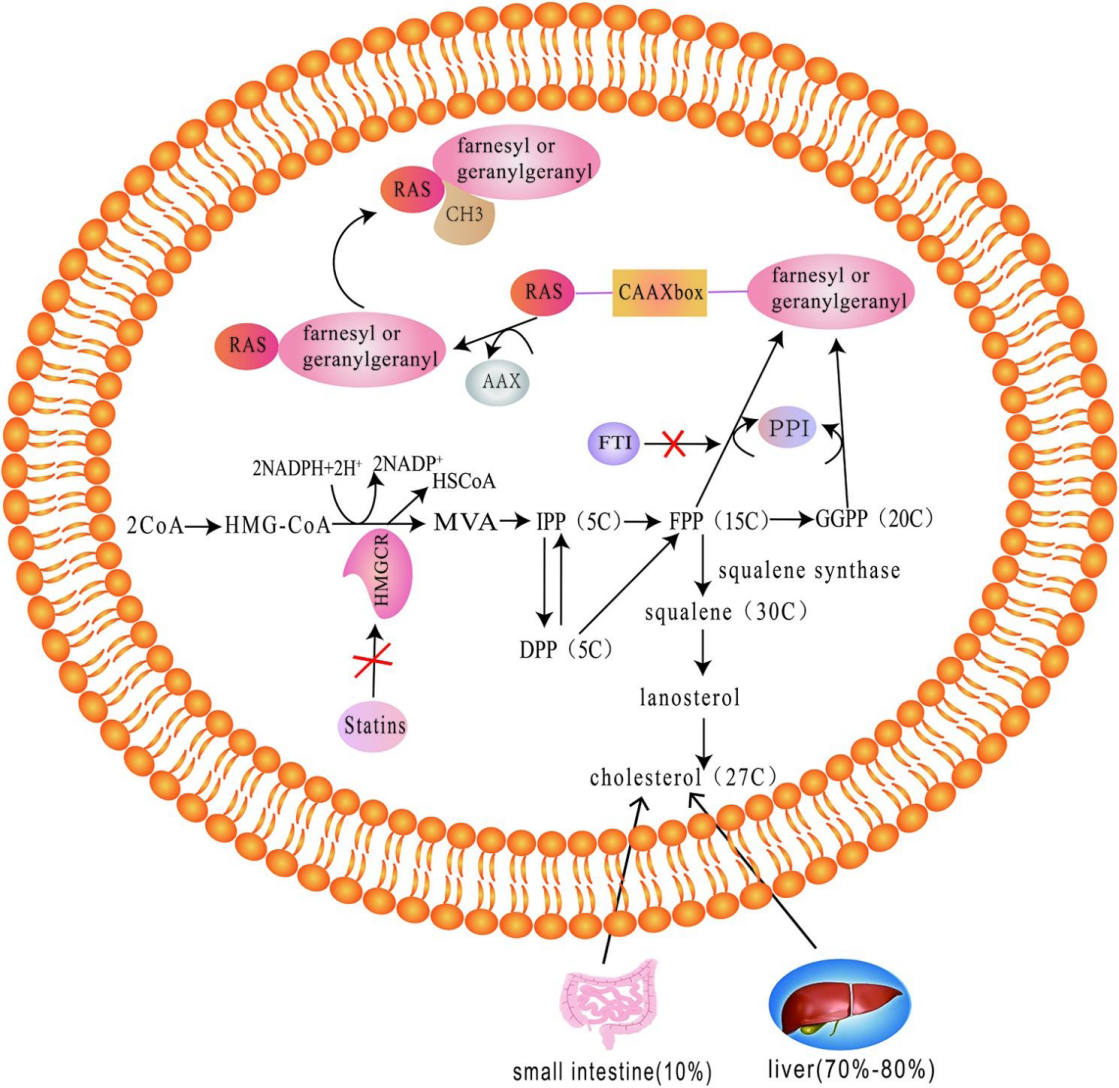
Schematic diagram of Isoprenylation modification steps and treatment of FTI and statins

Isoprenylation includes farnesylation and geranylgeranylylation, which refers to the process of adding farnesyl or geranyl to a specific cysteine residue near the C-terminal of the protein. The modification of isoprenylation can be divided into three steps: The first step is to attach the isoprene group derived from GGPP(geranylgeranyl pyrophosphate ) or FPP (farnesyl pyrophosphate) to the substrate of the protein under the catalysis of prenyltransferase. Then the geranylated or farnesylated protein is hydrolyzed and the downstream residues of the cysteine are hydrolyzed and removed. Finally, the carboxyl group of cysteine is methylated [106](Fig. 2).

### RAS proteins

The RAS oncogene family includes Kirsten RAS (KRAS), Harvey RAS (HRAS) and neuroblastoma RAS (NRAS), and ras oncogenic proteins include H-RAS, N-RAS, K-RAS4A and K-RAS4B, among which KRAS is the most widely studied [107]. The function of Ras protein depends on post-translational modification, which firstly requires farnesylation, that is, 15-carbon isopentyl farnesyl can be added to the cysteine sulfhydryl group of CAXX box at the carboxyl terminus of ras under the catalysis of farnesyl protein transferase. The protein then hydrolyzes AXX amino acids and methylates the carboxyl group of farnesylated cysteine [108].

#### Farnesyltransferase inhibitors (FTI) and geranylgeranylation inhibitor

Farnesyltransferase initiates the signaling of ras proteins attached to the plasma membrane [109]. FTI blocks the membrane localization of ras and its signaling pathway by inhibiting the isoprenylation of H-RAS rather than K-RAS, so H-RAS might be a therapeutic target for FTI-mediated reflex sensitization in patients with colorectal cancer [110, 111]. FTI exerts its anti-CRC effect by inhibiting the farnesylation of ras proteins and other peptides through complex mechanisms. On the one hand, FTI can act as an analogue of FPP, competitively inhibiting FPP binding to FTase and CAAX sequences of K-RAS; On the other hand, in the presence of FTI, the N- and K-RAS proteins of farnesyltransferase substrates may be geranylgeranylated [112, 113].

KT7595, a gliotoxin derivative, and the farnesyl transferase inhibitor manumycin derived from streptomyces can inhibit FPTase by inhibiting the farnesylation of the p21 and the activity of the p42MAPK/ERK2 in its downstream pathways, thus inducing apoptosis and inhibiting proliferation of gastrointestinal tumor cells. Notably, its mechanism does not involve the cholesterol synthesis pathway [114–116]. In addition, the inhibitor of geranylgeranylation, BAL9611, similar to manumycin, can inhibit geranylgeranylation of P21rhoA and then inhibit the phosphorylation of P42ERK2/MAPK, thereby inhibiting K-ras mutant colon cancer cells [117].

#### Statins

Statins, also known as HMG-CoA reductase inhibitors, work by inhibiting the mevalonate pathway by blocking the conversion of HMG-CoA to mevalonic acid, which is a rate-limiting step in cholesterol synthesis [118]. The final metabolites of the mevalonate pathway, farnesyl and geranyl, are substrates for the isoprenylation of Ras proteins [119]. The use of statins can reduce the risk of tumors, and multiple studies have found that statins may be associated with isoprenylation of Ras proteins, which modulates the pathway where Ras proteins are located and exert their therapeutic effects on tumors [120, 121].

In RAF-driven colorectal cancer, statins can inhibit the isoprenylation of Ras protein which is mediated by the downstream intermediates FPP and GGPP of the cholesterol synthesis pathway, and inhibit the activation of the PI3K/AKT pathway as well as Hippo pathway to inhibit the activity of cell growth [122].

KRAS protein is activated by isoprenylation, and activated Ras acts as the switch in EGFR and nucleus, which is critical for ras/raf/MAPK and PI3K/AKT pathways [113]. EGFR inhibitor cetuximab can be used as a superior chemotherapy drug for advanced colorectal cancer patients without KRAS mutation [123]. While for patients with KRAS-mutated metastatic colorectal cancer (mCRC) that abnormal activation of the RAS pathway leads to abnormal proliferation of tumor cells, statins may treat mCRC by inhibiting isoprenylation of ras. Notably, the use of statins such as simvastatin has not been found to enhance the efficacy of cetuximab in patients with mCRC [124, 125].

It was found that pravastatin inhibits the isoprenylation of growth-regulating proteins such as P21 ras to inhibit the synthesis of cholesterol [126, 127], however, it has been reported the efficacy of hydrophobic simvastatin is superior to hydrophilic pravastatin in the treatment of the majority of tumors including intestinal cancer, especially in the treatment of poorly differentiated cancer cells [128]. In addition, the apoptosis induced by lovastatin in human colon cancer cell line SW480 was also achieved by inhibiting the synthesis of cholesterol. The blocking of the isoprenylation of ras protein inhibits the activation of PI3 kinase, thus reducing the expression of anti-apoptotic molecule survivin and the apoptosis caused by the interference of survivin to RNA [129]. However, it has also been shown that lovastatin functions not by inhibiting farnesylation of the target protein, but by suppressing geranylgeranylation [130].

#### Other studies on ras proteins

PDEδ is a guanine nucleotide dissociation inhibitor (GDI-) -like solubilizing factor that can bind to the C-terminal farnesyl of KRAS to interfere with the localization and signaling pathways of ras, and influence the survival and proliferation of cells [131, 132]. Therefore PDEδ inhibitors might become a novel target for KRAS mutant colorectal cancer.

Curcumin extracted from the spice turmeric can take polyisoprenylated methylated protein methyl esterase (PMPMEase) as the target to reversibly inhibit the activity of PMPMEase, thus inducing the death of colorectal cancer cells [133]. Similar to statins, dehydroepiandrosterone (DHEA) inhibits the synthesis of cholesterol and the isoprenylation of P21 ras as well as its binding to cell membranes in colorectal cancer [134].

Monoterpenes, the non-nutritious dietary substances in citrus fruits and some plant essential oils, may exert antitumor effects by influencing the isoprenylation of other proteins except ras. For example, limonene, coriander oil and carvone can prevent patients from suffering from gastric cancer, and the mechanism may be that the inhibition of prenyltransferase affects the isoprenylation of substrates, meanwhile, it may also affect the activity of HMG CoA reductase [135].

### Others

Farnesyl pyrophosphates (FPPs) are fundamental enzymes in the synthesis of FPP during farnesylation [136]. As a substrate in the isoprenylation of proteins, FPP is the target of nitrogen-containing bisphosphonates [137]. In colon cancer cells, pamidronate (PAM) can inhibit the growth and induce apoptosis of tumor cells by inhibiting FPPs and blocking isoprenylation. Phosphatase of Regenerating Liver-3(PRL-3) relies on its CAAX sequence to undergo isoprenylation to enhance its ability to migrate and invade. The isoprenylation of PRL-3 alters its localization and the metastasis of gastric cancer cells, which leads to poor prognosis in patients [138]. In addition, C-isoprenylated flavonoids can promote apoptosis of colon cancer cells through increasing caspase activity [139].

## Citrullination

Citrullination, which was first discovered in rheumatoid arthritis [140], refers to the process that the positively charged arginine residues are deiminated into electrically neutral citrullinine residues under the catalysis of peptidyl arginine deiminases (PADIs or PADs). This process is accompanied by the removal of amino groups, so it is also known as peptide-arginine deamination [141]. Until now, the most widely studied proteins involved in citrullination are histones. In addition, keratin 8 (KRT8), heat shock protein 60 (HSP60), α-enolase (ENO1), tubulin β (TUBB), T cell receptor β chain (TCRβ) and vimentin (VIME) are also suggested to be citrullinated proteins in tumor cells [140, 142]. Citrullination is thought to play an important role in the development of cancers because it can affect the folding state and function of proteins and interfere with protein stability.

### PADIs

PADIs include PADI1-4 and PADI6, a total of five isoenzymes [143] (Table 2). In colon cancer patients, the anti-parasitic drug nitazoxanide (NTZ) targets PADI2 to stabilize PADI2, which increases the citrullination of β-catenin protein in cancer cells and blocks the Wnt/β-catenin signaling pathway, thereby inhibiting the growth of cancer cells [144]. In addition, it has been found that PADI2 can arrest the cell cycle in the G1 phase and inhibit the proliferation of colonic epithelial cells by catalyzing the citrullination, which may serve as a target for colon cancer treatment [145]. As the substrate of PADI4, the tumor suppressor protein ING4 binds to PADI4 for citrullination, which competitively destroys the affinity between ING4 and P53, affects the acetylation of P53 and inhibits the expression of P21 downstream in colorectal cancer [146]. In colorectal cancer cells, GSK3β binds to highly expressed PADI4 and is citrullinated at R344, promoting nuclear translocation of GSK3β from the cytoplasm to nucleus. Nuclear accumulation of GSK3β thus promotes ubiquitin-dependent proteasome degradation of CDKN1A, which is an important transcriptional target of p53 protein and regulatory factor of the cell cycle [147]. In addition, the components of ECM such as collagen are increased in cancers. In liver metastases of colorectal cancer, the citrullination of ECM proteins mediated by PADI4 can promote MET by enhancing the adhesion of cancer cells and reducing their migration and activity, which facilitates the growth of colorectal cancer cells that have metastasized to the liver [148].

**Table 2:** Information of PADs in gastrointestinal tumors

Isozyme	Cancer	Citrullinated protein	Effects	References
PAD2	Colorectal cancer	β-catenin	PAD2 can block Wnt signaling pathway, block cell cycle and inhibit the proliferation of cancer cells	[137, 138]
PAD4	Gastric cancer	CXCR2, KRT14,TNF-α, IL-8, Histone chip H3R26Cit and histone of NETs	PAD4 promotes the formation and metastasis of gastric cancer and affects tumor progression	[142–145]
PAD4	Colorectal cancer	ING4, GSK3β, ECM	PAD4 promotes tumor proliferation and migration, facilitates the growth of colorectal cancer cells metastasized to the liver	[139–141]

In gastric cancer, PADI4 with genetic susceptibility may up-regulate CXCR2, KRT14 and TNF-α by disrupting the p53 signaling pathway to regulate the microenvironment, angiogenesis, proliferation, differentiation and migration, apoptosis and inflammation of cells, thus promoting the occurrence of gastric cancer [149]. In addition, PADI4 has been found to promote the EMT and the migration of gastric cancer cells by up-regulating IL-8 [150]. The protein labeled with IPO-38, a highly sensitive and specific marker for the diagnosis of gastric cancer, is considered to be a member of the histone family and is regulated by citrullination mediated by PADI4 in gastric cancer [151]. On the one hand, PADI4 regulates the formation of H3R26Cit, which is the sequence with the strongest signal of citrullination, and the histone chip H3R26Cit could interact with H3K27me3 to form a complex, thus affecting the detective ability of IPO-38; On the other hand, PADI4 may promote histone citrullination of NETs, thereby influencing the progression of cells [152].

Because of its important role in the occurrence and development of tumors, PADI4 has been considered as a potential target for the treatment of gastrointestinal cancers. PADI inhibitors such as CI-amidine, GSK199, and GSK484 have been developed as therapeutic drugs for cancers, whereas CI-amidine is more effective because of its ability to irreversibly inactivate enzymes [153, 154]. The mechanism of CI-amidine is to increase the expression of miR-16 by inhibiting PADIs and decrease the expression of its target proteins cyclin D1 and E1, thereby reducing the proliferation of intestinal cancer cells and preventing tumorigenesis [155].

### Others

In patients with colorectal adenocarcinoma, the level of citrullinated antithrombin was found to be elevated. Considering that PADIs-mediated citrullination at Arg393 is associated with the lack of anticoagulant capacity, this phenomenon could partially explain the fact that patients do not have an increased risk of thrombosis [156].

## Succinylation

**Fig. 3:**
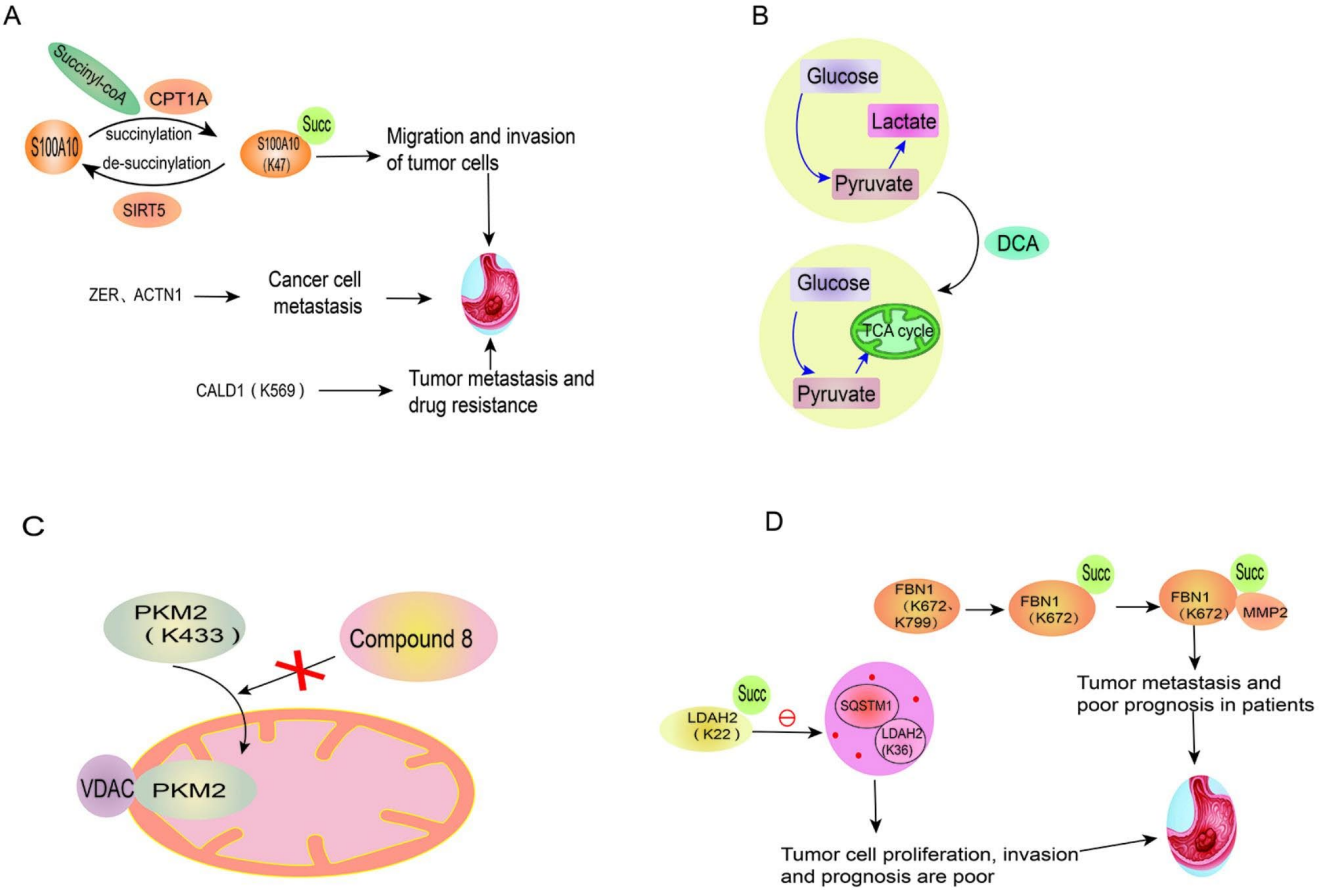
Role of Succinylation in gastric cancer. **A**. Schematic diagram of succinylation of proteins associated with the development and progression of gastric cancer. **B**. DCA reverses the Warburg effect. **C**. Schematic diagram of the mechanism of action of small molecule compound 8. **D**. Schematic diagram of succinylation of proteins associated with the prognosis of gastric cancer

First discovered in E. coli [157], succinylation refers to the transfer of succinyl from succinyl-CoA to a specific lysine residue of the target protein catalyzed by carnitine palmitoyl transferase 1 A (CPT1A), making positively charged lysine to be negatively charged [158, 159]. In addition, the de-succinylation is regulated by the de-succinylation enzyme SIRT5 [160] (Fig. 3). Interestingly, it has been shown that high levels of succinylation can also occur in tissues with high acyl-CoA (such as the heart and liver) by non-enzymatic mechanisms [161, 162]. Succinylation can regulate the activity of enzymes and metabolic pathways, thus playing an important role in cellular metabolism such as the tricarboxylic acid cycle and glycolysis [163]. A number of studies have shown that the level of succinylated proteins is closely related to the occurrence and development of gastrointestinal tumors.

### Tumorigenesis and development

S100A10 protein, a member of the calcium-binding cytoplasmic protein family [164], was found to be succinylated at K47, which inhibits its degradation in gastric cancer [165]. Stable S100A10 could thereby promote the migration and invasion of tumor cells. By identifying the lysine group of succinylation in gastric cancer tissues, it was found that ZER, ACTN1 and other down-regulated succinylated proteins were related to the activity of cytoskeletal, and may be closely associated with the metastasis of gastric cancer. Since the actin-binding protein caldesmon (CALD1) is a succinylated protein modified at K569 and is associated with metastasis and drug resistance of gastric cancer, it might serve as a critical marker of gastric cancer. In addition, the succinylation of the proteins is also regulated by LncRNAs such as H19, CCAT1 and GAS5 [166].

### Treatment

Warburg effect (also known as aerobic glycolysis) refers to the preference of malignant cells to obtain energy through glycolysis, even under aerobic conditions [167]. Among the enzymes in the TCA cycle, seven kinds of enzymes have been found to be involved in succinylation. Dichloroacetic acid (DCA), a pyruvate dehydrogenase (PDK) inhibitor can reverse the Warburg effect to normal phosphorylation, reduce the level of proteins involved in cell cycle, division and proliferation by inducing succinylation in mitochondria, and induce apoptosis [168]. Therefore, it is considered as a potential anti-tumor drug for the treatment of colorectal cancer [168].

Citrate synthase (CS), the rate-limiting enzyme of the TCA cycle, undergoes de-succinylation at the K393 and K395 sites after interacting with SIRT5. However, the highly succinylated CS at K393 and K395 inhibits colon cancer cell proliferation and migration, which provides a new target for therapy [169].

PKM2 translocated to the mitochondria is succinylated at K433 in the condition of glucose starvation, thereby binding to volt-dependent anion channel protein (VDAC) and stabilizing the VDAC protein, which increases mitochondrial permeability and contributes to tumor cell survival by inhibiting apoptosis through Bcl2. Therefore, small molecule compound 8, which could significantly reduce the activity of PKM2, was synthesized to inhibit the growth of tumors such as colon cancer by blocking the translocation of PKM2 [170].

### Prognosis

High expression of fibrinogen 1 (FBN1), a major component of extracellular matrix (ECM) [171], predicts poor prognosis in patients with gastric cancer. The succinylation of FBN1 at K672 inhibits its degradation by MMP2. In addition to promoting the proliferation of gastric cancer cells by activating TGF-β1 and PI3K/Akt pathways, the accumulated FBN1 can also promote the overexpression of MMP2 by increasing the level of HIF-1α, leading to extracellular matrix protein degradation and tumor metastasis [172].

Lactate dehydrogenase A (LDHA) is a key enzyme in aerobic glycolysis mediated by CPT1A [173]. Succinylation of LDAH at K222 affects the degradation of LDHA by binding to SQSTM1 in lysosomes, and the increased LDHA contributes to the proliferation and invasion of gastric cancer cells [9]. Thus, the level of succinylated LDHA is positively associated with poor prognosis in patients with gastric cancer.

## Other non-classical post-translational modifications

### Palmitoylation

Palmitoylation refers to the reversible modification of saturated fatty acid palmitate to the cysteine residues of the target protein [174]. The enzyme porcupine (PORC), located in the endoplasmic reticulum, is palmitoylated on serine residues [175]. Palmitoylation of PORC regulates the secretion of Wnt, and the Wnt receptor Frizzled7 (FZD7) is essential for the reverse conversion of EMT to MET, which allows colorectal cancer to metastasize to other organs [176]. Tumornecrosisfactor-Related Apoptosis-Inducing Ligand (TRAIL), a member of the TNF protein family [177], binds to trimer death receptors 4 (DR4) and 5 (DR5) to induce tumor cell apoptosis. Colorectal cancer cells resistant to oxaliplatin are more sensitive to TRAIL, and the mechanism is that the DR4 level is up-regulated due to increased palmitoylation of DR4 and increased metastasis to lipid rafts [178]. 17 β-estradiol (E2) receptors have two types, ERα and ERβ [179]. In colon cancer cells, the palmitoylation of ERα enables its localization on the plasma membrane, which plays a crucial role in the transcription of the cyclinD1 promoter and contributes to cell proliferation. In contrast to ERα, E2 induces an increase in ERβ levels in colon cancer cells and promotes ERβ palmitoylation through the activation of the P38/MAPK pathway, and palmitoylated ERβ in turn regulates the E2-induced apoptotic process and plays an anti-proliferation role [180, 181]. In colorectal cancer, palmitoylation may also play a role in internalization initiated by the binding of monoclonal antibody (mAb) A33 to antigen (A33) [182].

Immunotherapy resistance is often associated with mutations in IFN and MHC signaling genes [183]. Optineurin, the shared node of the two signaling pathways, affects the binding of AP3D1 to IFNGR1 (IFNγ receptor), which is palmitoylated at Cys122, thereby preventing IFNGR1 from being degraded by lysosomes and maintaining the integrity of the two signaling pathways [184, 185]. Therefore, in colorectal cancer, optineurin deficiency affects the expression of both pathways and reduces cellular immunity, resulting in drug resistance. Furthermore, 2-bromopalmitate activates anti-tumor immunity in colon cancer mice by inhibiting the palmitoylation of PD-L1 or silencing DHHC3, an enzyme required for the palmitoylation of PD-L1, which provides new ideas for overcoming PD-L1-mediated immune evasion [186]. In the treatment of colon cancer, 5-FU in combination with the antimalarial drug thioridazine or the CNS drug sertraline can improve the efficacy of anti-tumor drugs. Antimalarial drugs or CNS drugs target the PPT1 and inhibit the de-palmitoylation of PPT1 enzyme to suppress the mTOR signaling pathway, thus reducing the accumulation of anti-tumor drugs in the lysosome [187].

### Nitrosylation and nitration

The process of S-nitrosylation and nitration involves the addition of NO transfer to cysteine residues (S-nitrosylation) or tyrosine residues (nitration), which regulates the function of target proteins [188, 189].

NO donor anti-tumor drugs can induce cell apoptosis and inhibit cell metastasis without causing drug resistance in tumor cells [190]. Nitric oxide-donating aspirin (NO-ASA), a cancer preventive drug, promotes S-nitrosylation and tyrosine nitration of proteins in colon cancer cells by increasing NO. The mechanisms of its inhibition against colon cancer are complicated. NO donor inhibits the interaction of β-catenin with cysteine residues in the binding domain of TCF, thus inhibiting the S-nitrosylation modification of β-catenin and Wnt signaling pathway [191]. Also, NO donor inhibits the interaction of NF-kB with its homologous DNA oligonucleotides [192]. On the other hand, NO donor regulates the inactivation of NF-kB and promotes the degradation of β-catenin through tyrosine nitration in colon cancer [193]. It is worth noting that the level of nitrotyrosine is increased in colon cancer patients, and tyrosine nitration may predict the progression of cancer in patients [194]. In addition, peroxynitrite may promote the development of colorectal cancer by abnormally activating the NF-kB signaling pathway through the dual mechanisms of p38-dependent nitration and phosphorylation of IKB-α [195]. Besides, synthetic OA derivatives (SOADs) 4c can inhibit multidrug resistance (MDR) of drug-resistant cancer cells in colon cancer by inducing tyrosine residue nitration of ATP binding box (ABC) transporter protein, which is a novel treatment for drug-resistant colon cancer [196].

## Summary and future directions

Post-translational modifications (PTMs) of proteins regulate multiple functions as well as contribute to the occurrence and development of tumors. Apart from canonical protein post-translational modifications such as phosphorylation, ubiquitination, methylation, glycosylation and acetylation, recent investigations of non-canonical post-translational modifications suggest their close implication in occurrence, cell proliferation, invasion, metastasis, prognosis and treatment of gastrointestinal tumors. Various non-canonical post-translational modifications involve different signaling pathways, regulatory factors and enzymes in carcinogenesis and development.

Non-canonical post-translational modifications have been reported to be closely implicated in the development of gastrointestinal tumors. Studies suggested that non-canonical PTMs such as SENP2 mediated de-SUMOylation of N-myc, Mdm2 regulated neddylation of HuR, and PADI4 promoted histone citrullination of NETs contribute to the progression of gastrointestinal tumors. Furthermore, non-canonical PTMs including GLUT1-associated lncRNAs regulated SUMOylation of GLUT1 protein, PRL-3 isoprenylation mediated translocation, and succinylated CALD1 protein lead to metastasis of gastrointestinal tumors. Moreover, it has been unraveled that different non-canonical PTMs participate in the therapy response and drug resistance of gastrointestinal tumors.

Non-canonical PTMs of proteins diversify the proteome and significantly influence the activity, localization, and functional pathways of proteins. Similar to canonical PTMs, non-canonical PTMs are of profound significance in clinical diagnosis and treatment. SUMO and NEDD8 are ubiquitin-like peptides undergoing SUMOylation and neddylation, respectively. They are conjugated by a chain reaction of E1, E2, and E3 enzymes. Chemotherapeutic agents targeting the SUMOylation and neddylation processes are being developed, of which MLN4924 is the only E1 inhibitor in Phase II clinical trials. Despite the lack of specificity and low activity, several UBC9 inhibitors have been reported, including GSK145A, 2-D08, and spectectcinB1. The expression of E3 ligase is increased in tumors and is closely related to the poor prognosis of patients. Because of its specificity, two kinds of E3 ligase-based drugs, GDC-199 and nutlins, are used in clinical trials [197, 198].

Protein lipidation refers to the process by which various lipids and their metabolites bind to and modify proteins in cells. Lipidation not only regulates protein function, but also is closely related to metabolism and cell energy homeostasis. There are four common types of lipid modification, including myristoylation, palmitoylation, isoprenylation, and glycosylphosphatidylinositol anchoring [199]. Palmitoylation is the most common lipid modification and is therefore considered important in the treatment of cancer. For example, up-regulated fatty acid synthase (which catalyzes palmitate synthesis) in cancer has been identified as a therapeutic target. At present, researches on inhibitors of protein palmitoylation has been carried out but they lack specificity [200, 201]. Ras protein is a widely studied carcinogenic protein, and isoprenylation is closely related to ras protein. In addition to statins, which are already widely used in clinical, FTIs are one of the first signal transduction inhibitors to be tested for anti-tumor clinical properties. Although the results of Phase II clinical trials are not satisfactory and could not specifically select cancer cells, it still provides an idea for clinical treatment of RAS-related tumors and some non-neoplastic diseases, such as diabetic retinopathy and macular disease [202]. Isoprenyl carboxy methyltransferase inhibitors have been reported as chemotherapeutic agents targeting the ras signaling pathway, including cysmethynil, AdoHcy, AFC, and AGGC, but these inhibitors need to be further optimized before clinical use [203].

Citrullination of histones can be used as biomarkers and clinical therapeutic targets. For advanced cancer patients, CitH3 can be used to assess their inflammatory response and predict the occurrence of venous thromboembolism. By targeting citH3 and NET, compounds commonly used in herbal medicine can inhibit hematogenous metastasis of certain tumors. Given that citrullination is mediated by PAD, PAD inhibitors include reversible inhibitors (taxol, minocycline, streptomycin, SC97362, Inh-Dap) and irreversible inhibitors (NSC95397, streptonigrin, cl-amidine, F-amidine, O-cl) amidine, O-F-amidine, TDFA ) are investigated in order to overcome the defects of poor selectivity and low bioavailability [204, 205].

Studies on sumoylation, succinylation, neddylation, Isoprenylation, citrullination, nitrification, and nitrosation suggested promising application of non-canonical PTMs in clinical diagnosis, treatment and drug resistance of patients with gastrointestinal tumors. Other novel non-canonical PTMs such as ribosylation, formylation, O-GlcNAcylation, deamination, propionylation and butyrylation are currently poorly understood. In addition, specific mechanisms and clinical applications of non-classical PTMs have not been thoroughly studied. Further studies are required to elucidate the therapeutic potential of targeting non-classical PTMs to improve the treatment and prognosis of gastrointestinal tumors.

## Data Availability

All the data used in the manuscript are freely available online.
